# Sexually Dimorphic Neurotransmitter Release at the Neuromuscular Junction in Adult *Caenorhabditis elegans*

**DOI:** 10.3389/fnmol.2021.780396

**Published:** 2022-01-31

**Authors:** Zhenzhen Yan, Xinran Cheng, Yuansong Li, Zexiong Su, Yiwei Zhou, Jie Liu

**Affiliations:** Neuroscience Program, Department of Anatomy and Developmental Biology, Monash Biomedicine Discovery Institute, Monash University, Melbourne, VIC, Australia

**Keywords:** *C. elegans*, neuromuscular junction, synaptic plasticity, calcium sensitivity, sex differences

## Abstract

Sexually dimorphic differentiation of sex-shared behaviors is observed across the animal world, but the underlying neurobiological mechanisms are not fully understood. Here we report sexual dimorphism in neurotransmitter release at the neuromuscular junctions (NMJs) of adult *Caenorhabditis elegans*. Studying worm locomotion confirms sex differences in spontaneous locomotion of adult animals, and quantitative fluorescence analysis shows that excitatory cholinergic synapses, but not inhibitory GABAergic synapses exhibit the adult-specific difference in synaptic vesicles between males and hermaphrodites. Electrophysiological recording from the NMJ of *C. elegans* not only reveals an enhanced neurotransmitter release but also demonstrates increased sensitivity of synaptic exocytosis to extracellular calcium concentration in adult males. Furthermore, the cholinergic synapses in adult males are characterized with weaker synaptic depression but faster vesicle replenishment than that in hermaphrodites. Interestingly, T-type calcium channels/CCA-1 play a male-specific role in acetylcholine release at the NMJs in adult animals. Taken together, our results demonstrate sexually dimorphic differentiation of synaptic mechanisms at the *C. elegans* NMJs, and thus provide a new mechanistic insight into how biological sex shapes animal behaviors through sex-shared neurons and circuits.

## Introduction

Behavioral sex differences are observed in most, if not all animal species. Sexually dimorphic behaviors are the result of the presentation of specific neurons and synaptic connections exclusive in one sex, and/or sex-biased functional modulation of shared neurons in both sexes (Ngun et al., 2011; Portman, 2017; Choleris et al., 2018). Although the contribution of sexually dimorphic neuroanatomy in sex-specific behaviors (e.g., mating and courtship) has been extensively characterized (Ngun et al., 2011; Choleris et al., 2018), the neurobiological basis underlying sex-dependent physiological modulation of neurons and circuits common to both sexes, which ultimately generates differences in sex-shared animal behaviors, remains poorly understood.

The genetic model organism *Caenorhabditis elegans* has two biological sexes, XO male and XX hermaphrodite (a self-fertile female) (Herman, 2006; Barr et al., 2018). The complex and quantifiable behaviors, the fully described entire neuron lineage, as well as the completely mapped neural wiring diagram of both sexes, put *C. elegans* in a leading position to investigate the neurophysiological basis of sex differences in animal behavior (Hart, 2006; Hobert, 2010; Cook et al., 2019; Witvliet et al., 2021). Similar to other model organisms, *C. elegans* processes a number of sex-specific anatomical features in the nervous system, including male- and hermaphrodite-specific neurons and synaptic connections, which not only encode sex-specific behaviors, but also drive sexually dimorphic traits in behaviors developed in both sexes (Portman, 2017; Barr et al., 2018). For instance, the male-specific PHB-AVG synapse, which is eliminated in adult hermaphrodites during sexual maturation, facilitates mate searching in adult males (Oren-Suissa et al., 2016). In addition to sex-specific neurons and synaptic connections, sex-dependent physiological modulation of neurons presented in both sexes also encodes sexually dimorphic behaviors in *C. elegans*. For example, low expression of the food-associated chemoreceptor ODR-10 in AWA sensory neuron of well-fed males, one of the core sensory neurons in both sexes, promotes mate-search search (Ryan et al., 2014).

Sex differences in locomotion is one of the most prominent behaviors showing sexual dimorphism in *C. elegans*. Although the locomotion of males and hermaphrodites are both characterized as rhythmic sinusoidal movement, males move with a faster speed and a higher bending frequency and spend more time in exploration than hermaphrodites (Lipton et al., 2004; Barrios et al., 2008; Mowrey et al., 2014). Interestingly, this “hyperactive” locomotion in males is encoded by the lineally equivalent and almost morphologically identical motor system shared with hermaphrodites (Mowrey et al., 2014; Cook et al., 2019). As the fundamental and universal unit of the motor system, the neuromuscular junction (NMJ) in *C. elegans* plays a key role in the chemical signal transduction between motor neurons and muscle cells (Richmond, 2007). Excitatory acetylcholine (ACh) and inhibitory γ-aminobutyric acid (GABA) released from presynaptic motor neurons trigger muscle contraction or relaxation, respectively (Richmond, 2007). Neurotransmitter release at the NMJs in hermaphrodites has been well documented and is known to be modulated by multiple internal and external signals, such as the neuropeptide NLP-12 and the male pheromones (Hu et al., 2011; Qian et al., 2021). However, whether *C. elegans* motor neurons release neurotransmitters in a sexually dimorphic manner, and if so, to modulate behavioral differences between males and herapathites has not been addressed.

In this study, we aimed to investigate sex differences in worm locomotion at the synaptic level by conducing behavioral quantification, morphological analysis, and function characterization with the NMJs of both sexes. Our result showed that males exhibited enhanced locomotion velocities and superior bending angels, compared with hermaphrodite, and sex differences in worm locomotion were only observed between adult males and hermaphrodites. Correspondingly, the cholinergic NMJs exhibited adult-specific differences in synaptic structure between males and hermaphrodites. Further functional analysis of worm NMJs supported that adult males had increased neurotransmitter release and the higher calcium sensitivity of ACh and GABA release than hermaphrodites, while stronger synaptic depression and slower vesicle replenishment were observed in cholinergic NMJs in hermaphrodites. Interestingly, T-type calcium channels/CCA-1 was required for ACh release at the NMJs of males, but not hermaphrodites.

## Results

### Adult *Caenorhabditis elegans* Displays Sex Differences in Locomotion

**FIGURE 1:**
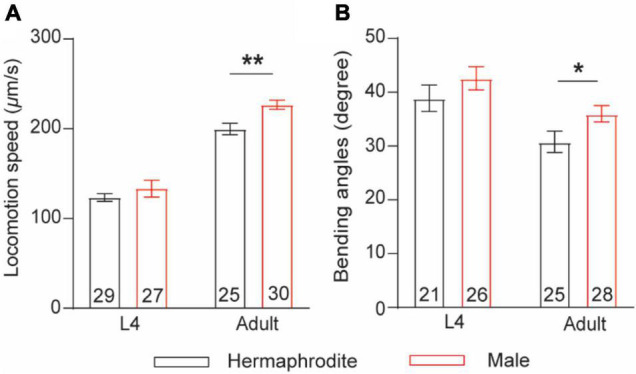
Adult *C. elegans* exhibits sex-specific difference in locomotion behaviors. **(A)** Bar graph of the locomotion speed of hermaphrodites and males at the larval stage L4 and adult stage (**p = 0.0015, *t*-test). **(B)** Bar graph of the bending angle of hermaphrodites and males in the larval stage L4 and adult stag (*p = 0.0416, *t*-test). Sample sizes (*n* numbers) are indicated in the bars of each graph. All data are represented as mean ± SEM.

The nematode *C. elegans* comprises two nature sexes: male (XO) and self-fertilization hermaphrodite (XX). To compare sex differences in locomotion behavior between male and hermaphrodite worms, we quantified the sinusoidal locomotion of *C. elegans* on the fresh *Escherichia coli* OP50-seeded NGM plates at both the larval stage L4 and the adult stage (Day 2) as described previously (Liu et al., 2013; Li G. et al., 2019). Our results were consistent with previous observations (Mowrey et al., 2014) showing that the locomotion of adult males was characterized with faster velocities and higher bending angels than that of adult hermaphrodites, while no obvious sex-related motor differences were observed at the larval stage L4 (Figure 1). These results support the adult-specific behavioral differences between male and hermaphrodite worms.

### Adult *Caenorhabditis elegans* Displays Sex-Specific Morphological Features of Cholinergic Neuromuscular Junction

**FIGURE 2:**
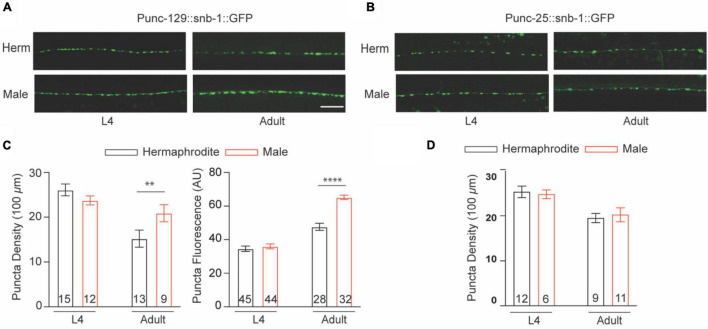
Cholinergic NMJs exhibit sex-specific difference in adult *C. elegans*. The distribution of *Punc-129:SNB-1:GFP* **(A)** and *Punc-25:SNB-1:GFP* **(B)** at NMJs of males and hermaphrodites at the larval stage L4 and adult stage. **(C)** Bar graph summarizing the *Punc-129:SNB-1:GFP* puncta density and fluorescence from males and hermaphrodites. **(D)** Bar graph summarizing the *Punc-25:SNB-1:GFP* puncta density from males and hermaphrodites. Sample sizes (*n* numbers) are indicated in the bars of each graph. All data are represented as mean ± SEM (^**^p < 0.01, ^****^p < 0.0001, *t*-test).

Caenorhabditis *elegans* sinusoidal movement results from the alternating contraction and relaxation of body wall muscle cells, which are triggered by ACh and GABA released at the NMJs, respectively. The NMJ is a specialized synapse where motor neurons release chemical signals to innervate muscle activation. We therefore asked whether the synaptic structure of the NMJ also exhibits sex differences between males and hermaphrodites during development. The excitatory cholinergic and inhibitory GABAergic NMJs along the ventral nerve cord were observed with the integrated strains *nuIs152* and *juIs1*, in which GFP-targeted synaptobrevin (SNB-1:GFP) driven by the promoter *unc-129* and *unc-25*, respectively (Hallam and Jin, 1998; Ch’ng et al., 2008). Imaging analysis showed the average number of cholinergic puncta *per* 100 μm in adult males and the fluorescent intensity were significantly higher than that in adult hermaphrodites, although SNB-1:GFP puncta in GABAergic synapses did not display obvious sex-dependent differences (Figure 2). Since SNB-1 is presynaptic vesicle-associated protein required for versicle release, these results suggested that adult males host more presynaptic vesicles at NMJs than adult hermaphrodites. Because sex differences in locomotion were only observed between adult males and hermaphrodites, rather than worms at the last larval stage L4, we next investigated whether sex differences in the synaptic architecture at the NMJs are also developmental stage-dependent. We found that males and hermaphrodites at the L4 stage processed a similar number of SNB-1:GFP puncta in the cholinergic synapses along the ventral nerve cord (Figure 2C). Taken together, these results suggest that *C. elegans* only processes adult-specific sex differences in the excitatory cholinergic NMJs.

### Neurotransmitter Release at the Neuromuscular Junctions of Adult Males Has an Enhanced Sensitivity to Extracellular Calcium

**FIGURE 3:**
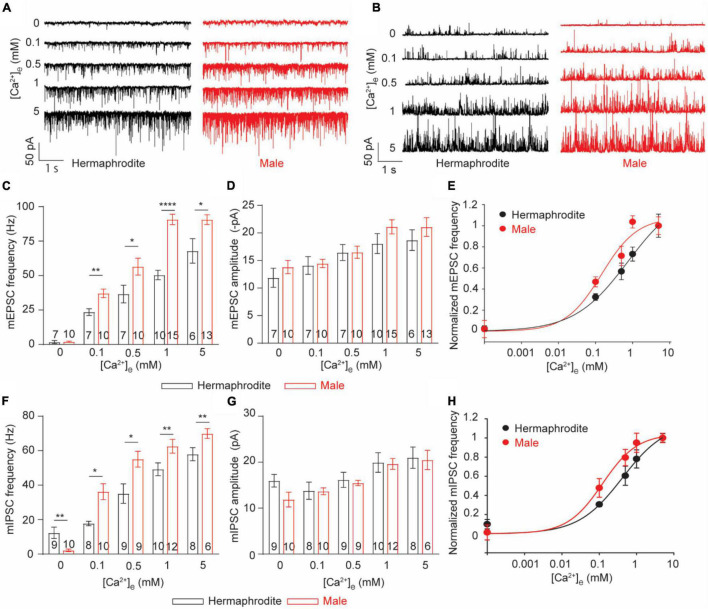
*C. elegans* males exhibit enhanced Ca^2+^ sensitivity of ACh and GABA release at the NMJs. **(A,B)** Sample traces of mEPSC recorded from the NMJs of adult hermaphrodites and males under distinct extracellular calcium ([Ca^2+^]_e_) (0, 0.1, 0.5, 1, and 5 mM). Bar graph of the frequency **(C)** and the amplitude **(D)** of mEPSC recorded from the NMJs of adult hermaphrodites and males under distinct [Ca^2+^]_e._ The Ca^2+^ sensitivity of spontaneous ACh release **(E)** and spontaneous GABA release **(H)** greatly enhanced in adult males. The normalized frequency of mEPSC and mIPSC is plotted as a function of [Ca^2+^]_e._ The plots are fitted by the Hill equation with difference EC50 (mEPSC: 0.1450 males vs. 0.8569 hermaphrodites; mIPSC: 0.1034 males vs. 0.4819 hermaphrodites). Bar graph of the frequency **(F)** and the amplitude **(G)** of mIPSC recorded from the NMJs of adult hermaphrodites and males under distinct [Ca^2+^]_e._ Sample sizes (*n* numbers) are indicated in the bars of each graph. All data are represented as mean ± SEM (*p < 0.05, **p < 0.01, ****p < 0.0001, two-way ANOVA followed by Bonferroni’s *post hoc* tests).

Sex-specific anatomical features of the NMJ of adult animals promoted us to investigate the function of the NMJ in adult males and hermaphrodites. Recording postsynaptic currents in muscle cells offers a reliable and comprehensive approach for the functional evaluation of the NMJs in different species (Liu et al., 2013). With the specific intracellular solution and the bath solution for whole-cell voltage-clamp recording from muscle cells, spontaneous ACh and GABA release at the *C. elegans* NMJs were recorded as miniature excitatory postsynaptic currents (mEPSC) and miniature inhibitory postsynaptic currents (mIPSC) at a holding potential of −60 or 0 mV, respectively (Dong et al., 2015). We first quantified the spontaneous vesicular ACh and GABA release at the NMJs with 1 mM Ca^2+^ in the bath solution, which was used in the following experiments unless otherwise specified. Compared with adult hermaphrodites, adult males exhibited increased frequencies of mEPSC and mIPSC without changes in mini amplitudes (Figures 3A–G). At *C. elegans* NMJs, the amplitude of mEPSC and mIPSC depend on the quantal content of presynaptic vesicles and the physiological function of postsynaptic reporters at NMJs, while the frequency reflects the presynaptic activity at NMJs. The enhanced mini frequency in adult males therefore was mostly like result from an increase in presynaptic vesicle release rather than a difference at postsynaptic receptors at the NMJs between adult males and hermaphrodites.

Neurotransmitter release at *C. elegans* NMJs is a classic calcium-dependent vesicle exocytosis, which is tightly controlled by the conserved SNARE complex, including the Ca^2+^ binding protein UNC-13 and synaptotagmin (Richmond, 2007; Sudhof, 2012). Extracellular Ca^2+^ modulates neurotransmitter release in a concentration-dependent manner in many types of neurons, including the motor neuron of adult hermaphrodites (Sudhof, 2012; Liu et al., 2018). The observed sex differences in the mini frequency recorded with 1 mM extracellular Ca^2+^ imply different calcium sensitivities of the neurotransmitter release at the NMJs between males and hermaphrodites. Therefore, we next examined the calcium sensitivity of neurotransmitter release at the NMJs by recording mEPSC and mIPSC of both sexes with bath solutions containing difference Ca^2+^ (0, 0.1, 0.5, or 5 mM). In agreement with previous studies, the frequency and amplitude of both mEPSC and mIPSC of adult hermaphrodites were significantly increased under the bath solution containing high levels of Ca^2+^ (Figure 3; Liu et al., 2018). The extracellular Ca^2+^-dependent increase in the frequency and the amplitude of both mEPSC and mIPSC was also observed at the NMJ of adult males, supporting that the motor neurons in both males and hermaphrodites release ACh and GABA in an extracellular Ca^2+^-dependent manner (Figure 3). However, we found a left shift of the extracellular Ca^2+^ and the relative mini frequency relation in male worms, and the relation was fitted with the Hill equation with a lower EC 50, compared to that in adult hermaphrodites (Figures 3E,H), indicating an enhanced Ca^2+^ sensitivity of neurotransmitter release at the NMJ of adult males. It should be noted that the Ca^2+^-independent GABA spontaneous release at the NMJ was observed in adult hermaphrodites, not adult males (Figure 3F). Collectively, our findings suggest the spontaneous neurotransmitter release at the NMJ of both sexes is characterized with an extracellular Ca^2+^-dependent exocytosis, but the neurotransmitter release in adult male worms has an enhanced sensitivity to extracellular Ca^2+^.

### Sex Differences in the Neurotransmitter Release at the Neuromuscular Junctions Are Developmental Stage Dependent

**FIGURE 4:**
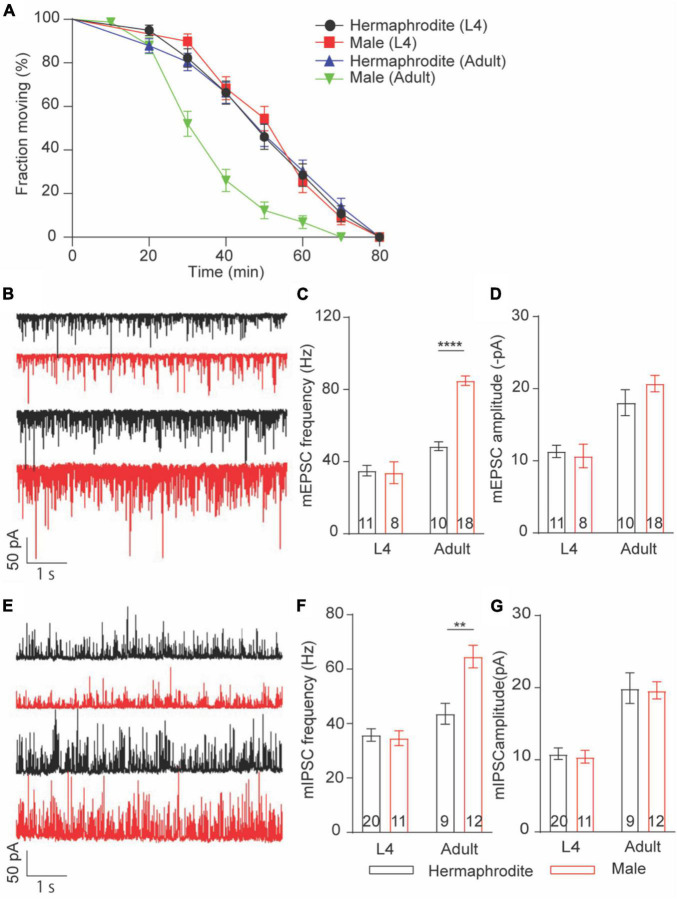
Sex-specific calcium sensitivities of neurotransmitter release at the NMJs are developmental stage-dependent. **(A)** The time course of 1 mM aldicarb-induced paralysis with hermaphrodites and males at the larval stage L4 and adult stage. **(B)** Sample traces of mEPSC recorded from the NMJs of hermaphrodites and males at the larval stage L4 and adult stage. Bar graph of the frequency **(C)** and the amplitude **(D)** of mEPSC recorded from the NMJs of hermaphrodites and males at the larval stage L4 and adult stage. **(E)** Sample traces of mIPSC recorded from the NMJs of hermaphrodites and males at the larval stage L4 and adult stage. Bar graph of the frequency **(F)** and the amplitude **(G)** of mIPSC recorded from the NMJs of hermaphrodites and males at the larval stage L4 and adult stage. Sample sizes (*n* numbers) are indicated in the bars of each graph. All data are represented as mean ± SEM (**p < 0.01, ****p < 0.0001, *t*-test).

Sex differences in locomotion were only found in adult worms. We thus asked whether sex differences in the Ca^2+^ sensitivity of vesicle exocytosis at the NMJs are developmental stage dependent. To address this question, we first evaluated the *in vivo* acetylcholine release at the NMJs with an aldicarb-induced paralysis assay (Hu et al., 2011; Qian et al., 2021). Aldicarb inhibits acetylcholinesterase, which in turn causes the sustained activation of muscular ACh receptor and leads to a hypercontractive paralysis *via* the accumulation of ACh at NMJs (Hu et al., 2011). Consistent with the sex difference in locomotion and ACh release at the NMJ of adult animals, the rate of aldicarb-induced paralysis in adult males was significantly faster than that of hermaphrodites (Figure 4A). By sharp contrast, the paralysis rate is comparable between L4 males and hermaphrodites (Figure 4A). These behavior analyses indicated that sex differences in the neurotransmitter release at the NMJs are developmental stage dependent. To provide additional evidence, we next examined the spontaneous neurotransmitter release at the NMJ of L4 and adult animals. As our expectation, the average frequency and amplitude of both mEPSCs and mIPSCs of males were generally comparable to those of hermaphrodites at the larval stage L4, although the sex-specific difference in the mini frequency was observed again in adult animals (Figures 4B–G). Altogether, our results indicate that sex differences in the neurotransmitter release at the NMJs depend on the developmental stage of *C. elegans*. The strong development correlation among the changes of locomotion, the alternation of synaptic connections, and the neurotransmitter release at the NMJs indicates the mechanistic link among these properties in *C. elegans*.

### Cholinergic Synapses in Adult Males Exhibit Slower Depression and Faster Replenishment Than That in Hermaphrodites

**FIGURE 5:**
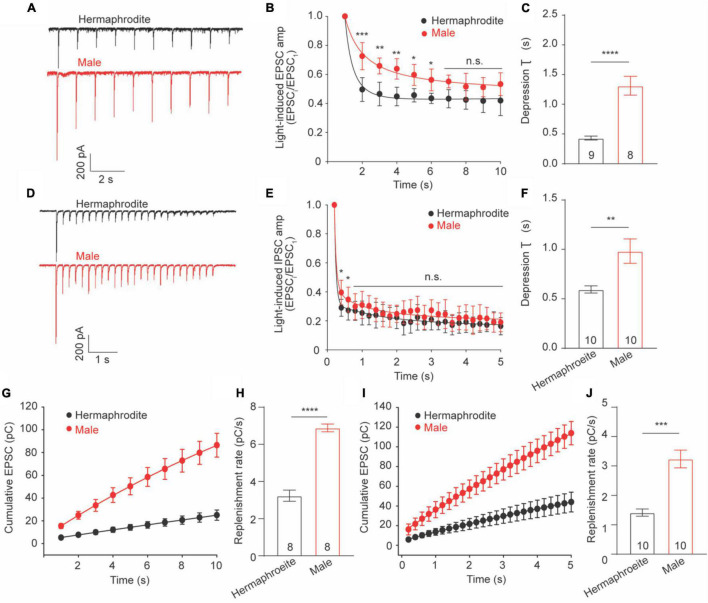
Synaptic depression and replenishment at the NMJs differ between adult hermaphrodite and male *C. elegans*. Synaptic depression and recovery were investigated by applying a 1 or 5 Hz stimulus train with worms expressing ChR2 in cholinergic motor neurons. Sample traces of 1 Hz **(A)** and 5 Hz **(D)** light train stimulus-evoked EPSC. Normalized evoked EPSC amplitude during 1 Hz **(B)** and 5 Hz **(E)** stimulation train. Bar graph of the depression index of the normalized amplitude of evoked EPSC in response to 1 Hz **(C)** and 5 Hz **(F)** stimulation train. Cumulative charge of evoked EPSC during 1 Hz **(G)** and 5 Hz **(I)** stimulation train. Bar graph of the replenishment rates during 1 Hz **(H)** and 5 Hz **(J)** stimulation train. Sample sizes (n numbers) are indicated in the bars of each graph. All data are represented as mean ± SEM (*p < 0.05, **p < 0.01, ***p < 0.001, ****p < 0.0001, n.s. indicates non-significant. *t*-test).

Sex difference in extracellular Ca^2+^ sensitivity of the neurotransmission at the NMJs indicates different plasticity properties of the NMJs between adult males and hermaphrodites. Previous research demonstrated that synaptic plasticity at the cholinergic NMJs of adult hermaphrodites was characterized with synaptic depression in response to a train stimulus in motor neurons (Liu et al., 2009; Li et al., 2021). We therefore sought to investigate whether biological sex affects synaptic plasticity at the cholinergic NMJs. To address this question, we recorded the light-evoked postsynaptic excitatory currents (evoked EPSCs) in muscle cells of worms zxIs6 expressing channelrhodopsin, a light-gated cation channel, in cholinergic motor neurons (Liu et al., 2009). For the evaluation of synaptic plasticity, the amplitude of evoked EPSCs was measured and normalized to the first evoked amplitude. Application of a stimulus train at 1 or 5 Hz led to the decreased amplitude in response to repeated 3 ms light exposures in both adult males and hermaphrodites, supporting the synaptic depression of the cholinergic NMJs in both sexes (Figures 5A–E). However, the relative amplitude of the evoked EPSCs from the second to the sixth stimulus in 1-Hz train of adult males, as well as the second and the third evoked EPSCs in 5-Hz stimulus, was significantly higher compared to the corresponding responses in hermaphrodites (Figures 5B,E). The depression rate of cholinergic synapses in adult males was significantly slower than that of hermaphrodites (Figures 5C,F). It supports that the ACh release at the NMJs in adult males is characterized with a weaker slow depression under both 1- and 5-Hz stimulus compared with adult hermaphrodites.

Weaker depression in male worms may arise from a faster replenishment rate of synaptic vesicles in the presynaptic terminals. We thus calculated the replenishment rate by fitting the slope of the cumulative amplitude of evoked EPSCs as previously described (Li L. et al., 2019; Li et al., 2021) and found that adult male worms displayed faster replenishment rates in response to both 1− and 5-Hz stimulus compared to adult hermaphrodites (Figures 5G–J). Taken together, our results demonstrated the synaptic depression at the cholinergic NMJs of both adult males and hermaphrodites, and revealed weaker depression and faster replenishment rates of vesicle release at the cholinergic NMJ in adult males.

### CCA-1 Plays a Male-Specific Role in Acetylcholine Release at the Cholinergic Neuromuscular Junctions

**FIGURE 6:**
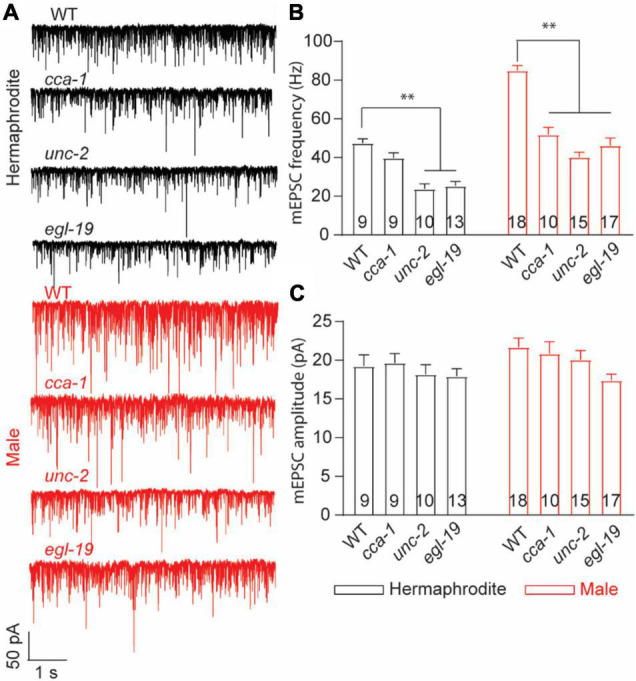
T-type calcium channel CCA-1 plays different roles in ACh release between adult hermaphrodites and males. **(A)** Sample traces of mEPSC recorded from the NMJs of adult WT and calcium channel mutants. Bar graph of the frequency **(B)** and the amplitude **(C)** of mEPSC recorded from adult WT and calcium channel mutants. Sample sizes (*n* numbers) are indicated in the bars of each graph. All data are represented as mean ± SEM (*p < 0.05, ^**^p < 0.01, two-way ANOVA with Dunnett test).

Synaptic vesicle exocytosis is directly triggered by the influx of Ca^2+^ through voltage-gated calcium channels (VGCCs). In *C. elegans*, *egl-19*, *unc-2*, and *cca-1* encode the central pore-formation α1-subunit of the CaV1 (L-type), CaV2 (R-, N-, and P/Q-type), and CaV3 (T-type) channels, respectively (Schafer and Kenyon, 1995; Lee et al., 1997; Steger et al., 2005). Different behavioral defects were reported with the mutant hermaphrodites in *egl-19*, *unc-2*, or *cca-1*, such as the uncoordinated movement with *unc-2* mutants (Schafer and Kenyon, 1995). Previous research emphasized the essential role of EGL-19 and UNC-2 in spontaneous neurotransmitter release at the NMJ of adult hermaphrodites (Tong et al., 2017; Liu et al., 2018). To identify the calcium channels responsible for the sex-specific neurotransmitter release at the male NMJs, we compared the frequency and the amplitude of mEPSC recorded from three VGCC mutants with that of WT worms. In line with previous research (Tong et al., 2017; Liu et al., 2018), the decreased frequency of mEPSCs was found in *unc-2* null mutants and *elg-19* loss-of-function mutant hermaphrodites, but not *cca-1* null mutants (Figure 6). Interestingly, all three mutant males exhibited decreased frequencies of mEPSC (Figure 6B), supporting that CCA-1, UNC-2, and EGL-19 are required for the spontaneous ACh release at the male NMJs. It should be noted that no significant changes were found in the amplitude of mEPSC in three VGCC mutant animals (Figure 6C). Changes in the mEPSC amplitude reflect the physiological alternations in the ACh quantal release from each presynaptic vesicle and the physiological function of postsynaptic muscle receptors. Three VGCC mutants therefore had unchanged quantal size of presynaptic vesicles and unaltered function of postsynaptic receptors in muscle cells, compared to wild-type animals. Collectively, these results confirm the function of EGL-19 and UNC-2 in the neurotransmitter release at the worm NMJs and suggested that CCA-1 plays a male-specific role in the spontaneous ACh release at the NMJs.

### Muscle Cells of Males and Hermaphrodites Exhibit Different Acetylcholine-Evoked Responses

**FIGURE 7:**
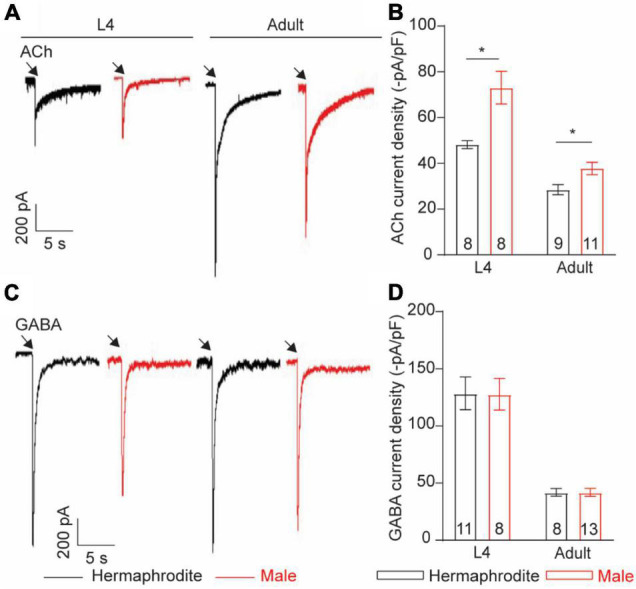
Muscle cells exhibit sex-specific difference in ACh current intensity. Sample traces of body-wall muscle currents evoked by 1 mM ACh **(A)** and 1 mM GABA **(C)** in hermaphrodites and males at the larval stage L4 and adult stage. Bar graph of ACh-evoked currents **(B)** and GABA-evoked currents **(D)** in body-wall muscle cells. Sample sizes (*n* numbers) are indicated in the bars of each graph. All data are represented as mean ± SEM (*p < 0.05, *t*-test).

Neurotransmitter released from motor neurons of *C. elegans* stimulates the postsynaptic receptors of muscle cells to direct muscle activities. Alternation in postsynaptic receptors has been linked with the locomotion differences in *C. elegans* (Richmond and Jorgensen, 1999). Sexual modification of muscle cells modulates the wave propagation speed in *C. elegans* (Mowrey et al., 2014), implying the sex-specific modulation of the muscular receptors in *C. elegans*. To check sex-specific properties of the ACh and GABA receptors in muscle cells, we recorded the ACh- and GABA-evoked muscle currents with drug puff application. We found that the intensity of the ACh-evoked inward currents in adult males was significantly higher than that in adult hermaphrodites (Figures 7A,B). Interestingly, the increased intensity of the ACh-evoked inward currents was also observed in males at the larval stage L4 (Figures 7A,B). By sharp contrast, muscle cells of adult males and hermaphrodites displayed similar responses when exogenous GABA was applied (Figures 7C,D). Taken together, our results support the sex-specific modulation of the muscle ACh receptors in muscle cells of *C. elegans*.

## Discussion

How biological sex generates sexual dimorphism of the human brain and drives different behaviors between males and females has been recognized as one of the fundamental questions of neuroscience since the ground-breaking study of Raisman and Field (1973). However, understanding the neurophysiological basis underlying sexually dimorphic behaviors in humans is extremely challenging, given the complexity of the brain and the ethical considerations in human research. The nematode *C. elegans*, a genetic model organism with the complete cell lineage and the fully mapped connectomes, also exhibits sex-dependent differences in multiple behaviors, such as male-specific copulation and sex-biased olfaction (Sulston et al., 1983; Portman, 2017; Barr et al., 2018; Cook et al., 2019). Sex differences in worm behaviors, similar to those of humans, result from the sex-related physiological differentiations in the nervous system at the level of neurons, circuits, and synapses (Portman, 2017). *C. elegans* sinusoidal locomotion is the pronounced worm behavior characterized with different physiological traits between males and hermaphrodites (Mowrey et al., 2014). Our studies demonstrate that the development-dependent sexual dimorphism at the level of synaptic structure, synaptic communication, and animals’ behavior (model Supplementary Figure 1), which build upon the linearly equivalent and anatomically identical motor unit shared with both sexes. Studying *C. elegans* locomotion therefore provides novel insights for connecting the sexually dimorphic modulation of synaptic transmission to behavior, despite the great diversity of the neuroanatomy in animals.

The neurotransmission at *C. elegans* NMJs directly correlates with the locomotion speed of *C. elegans* (Liu et al., 2013). Our studies show that the enhanced neuromuscular transmission and the higher locomotion speed in males are only observed at adult stages, supporting that sexually dimorphic synaptic organization and function drives sex differences in the motor performance between adult males and hermaphrodites. The critical role of the sex-specific synaptic strength in sexually dimorphic behaviors has been long appreciated in many animal species, including humans (Choleris et al., 2018; Hyer et al., 2018). For example, male-specific properties of long-term potentiation (LTP) in hippocampal field CA1 result in better motor and spatial ability in males (Wang et al., 2018). Sex-specific synaptic strength in Mammals normally stems from sex-dependent adult neurogenesis and synaptogenesis, as well as sex-specific response to hormones. In *C. elegans*, all male-specific neurons differentiate in the last larval stages L4, and some of these neurons are connected to postsynaptic motor neurons by chemical synapses (Oren-Suissa et al., 2016; Cook et al., 2019). Furthermore, multiple sex-specific synaptogenesis, such as male-specific synaptic connection between AVG and VD13, exclusively display at the adult stage (Oren-Suissa et al., 2016). Whether these development-dependent and sex-specific features in the nervous system also contribute to sexually dimorphic synaptic transmission in adult worms requires further investigation. In addition to adult-specific sex differences in neuroanatomy, recent research shows that male-specific pheromones modulate the locomotion speed of hermaphrodites by promoting the ACh release at the *C. elegans* NMJs (Qian et al., 2021). Because males used in our studies were grown on “male + hermaphrodite” plates, quantifying the synaptic function with males cultured on “male” only plates will evaluate the potential role of hermaphrodite-specific signals in the synaptic morphology and function in males.

Our research reveals an unexpected role of the *C. elegans* T-type voltage-gated calcium channel/CCA-1 in ACh release at the male NMJs. Voltage-gated calcium channels (VGCCs) play pivotal roles in the timing and strength of synaptic neurotransmitter release (Carbone et al., 2014; Dittman and Ryan, 2019; Dolphin and Lee, 2020). VGCCs are divided into two groups: high-voltage activated calcium channels Cav1 (L-type) and Cav2 (N-, P/Q-, R-type) and low-voltage activated calcium channel Cav3 (T-type). Although Cav2 channels dominantly control the neurotransmitter release at most synapses, increasing evidence supports that Cav1 and Cav3 actively regulate presynaptic calcium influx and vesicle release at specific synapses (Dolphin and Lee, 2020). Specifically, Cav3 channels control basal exocytosis above the resting membrane potential at various neurons, such as glutamate release in cortical neurons and GABA release at hippocampal perisomatic interneurons (Dolphin and Lee, 2020). In *C. elegans*, *egl-19*, *unc-2*, and *cca-1* encode the pore-forming α1 subunit of Cav1, Cav2, and Cav3, respectively (Schafer and Kenyon, 1995; Lee et al., 1997; Steger et al., 2005). In hermaphrodites, UNC-2 and EGL-19 control spontaneous ACh release at the NMJs in a cell-autonomous manner, while *cca-1* mutant has normal neurotransmitter release at the NMJs (Tong et al., 2017; Liu et al., 2018). Our research demonstrates that CCA-1, working together with UNC-2 and EGL-19, modulates the ACh release at the male NMJs. The sexual dimorphism of CCA-1 function in neuromuscular transmission could stem from either sex differences in the internal state of motor neurons or male-specific expression and modulation of CCA-1. Previous research shows that masculinization of the core locomotor circuitry (motor neurons and command interneurons) does not induce male-like locomotion in hermaphrodites, but masculinization of the entire nervous system enhances the locomotion speed of hermaphrodites (Mowrey et al., 2014). Although it cannot fully rule out the possibility that sexually dimorphic physiology of motor neurons generates the sex-different function of CCA-1 in vesicle release, a likelier explanation for male-specific function of CCA-1 is that male-specific signals modulate CCA-1 in motor neurons in a cell non-autonomous manner. Further studying in CCA-1 will provide a novel insight into the molecular mechanisms underlying sexually dimorphic behaviors in *C. elegans*.

## Materials and Methods

### Strains of *Caenorhabditis elegans*

The *C. elegans* strains were grown and maintained on nematodes growth medium (NGM) plates at 20°C (Stiernagle, 2006). The following strains were obtained from the Caenorhabditis Genetics Centre (CGG): Wide Type (Bristol strain N2), *nuIs152[Punc-129:GFP:SNB-1]*, *juIs1[Punc-25:snb-1:GFP]*, *cca-1(ad1650), unc-2(e55), egl-19(n580), and zxIs6 [Punc-17:ChR2(H134R):YFP]*. All males were generated by heat shock and the mutants were confirmed by Sanger sequencing.

### Behavioral Analysis

Worm tracking and analysis were performed as previously described with minor modifications (Liu et al., 2013; Li G. et al., 2019). Day 2 adult animals were transferred onto NGM plates seeded with 50 μL fresh *E. coli* OP50 (6–8 worms per plate) for behavioral analysis. 5 min videos of multiple animals were captured at 3.75 Hz frame rate with WormLab imaging system (MBF Bioscience, United States). A standard ruler was used to set the measurement scale for videos. Locomotion characteristics were analyzed using WormLab software (MBF Bioscience, United States).

### Drug Assays

Aldicarb-induced paralysis assays were conducted following the method previously described (Hu et al., 2011; Qian et al., 2021). Nematode growth media (NGM) plates containing 1 mM aldicarb (Sigma, United States) were seeded with 50 μL fresh *E. coli* OP50 and allowed to dry for 30–60 min at room temperature before the experiment. Animals (blinded for genotype) were transferred to aldicarb-supplemented plates and were individually touched on the posterior body to assess movement every 10 min. Animals were considered as paralyzed when no main body or head movement was detected.

### Fluorescence Imaging

All quantitative imaging was captured on a Nikon C1 upright confocal, using a CFI Plan Apochromat VC 100X oil objective and a DS-Fi3 camera (Roper, Trenton, NJ, United States). Worms were paralyzed with 20 mM sodium Azide in M9 buffer and mounted on 5% agar pads for imaging. For ventral nerve cord (VNC) imaging, in which the ventral cord was oriented toward the objective, worms were imaged in the region midway between head and vulva. Stacks of confocal images were used. Images were processed using ImageJ software, and the number of synapse clusters was counted manually. All intensity measurements were performed as previously described (Fry et al., 2014). The background fluorescence signal was subtracted before intensity analysis, and fluorescence intensity was measured in arbitrary units (AU).

### Retinal Feeding

NGM plates (60 mm) were seeded with 250 μL fresh OP50 mixed with 5 μL 100 mM all-trans retinal (Sigma) as previously described (Piggott et al., 2011). Seeded retinal plates were stored in the dark at 4°C for less than 1 week until used. L4 transgenic hermaphrodites and males were transferred to retinal plates and grown in the dark for additional 48 h at room temperature.

### Electrophysiology

Electrophysiology was conducted on dissected *C. elegans* using a protocol described by Ward et al. (2008). Whole-cell patch-clamp recordings were performed under an Olympus microscope (BX51WI) with an EPC-10 amplifier and the Patchmaster software (HEKA). Current data were filtered at 2 kHz and sampled at 20 kHz. Series resistance and membrane capacitance were both compensated. Recording pipettes were pulled from borosilicate glass capillaries (BF150-86-10, Sutter Instruments, United States) to a resistance of 3–5 MΩ. An L4 or Day 2 worm was glued on a sylgard-coated coverslip in bath solution using a medical grade, cyanoacrylate-based glue (Dermabond Topical Skin Adhesive, Ethicon). A small piece of cuticle on the worm body was carefully cut and glued down to the coverslip to expose muscle cells for recording. The regular bath solution contains (in mM) 140 NaCl, 5 KCl, 5 MgCl_2_, 1 CaCl_2_, 11 glucose, and 10 HEPES (330 mOsm, pH adjusted to 7.3). The pipette solution contains (in mM) 135 CH_3_O_3_SCs, 5 CsCl, 5 MgCl_2_, 5 EGTA, 0.25 CaCl_2_, 10 HEPES, and 5 Na_2_ATP (325 mOsm, pH adjusted to 7.2 using CsOH). Body wall muscle cells were clamped at −60 mV and 0 mV for recording mEPSC and mIPSC, respectively. To record ACh- and GABA-induced currents in muscle cells, the pipette solution contains (in mM) 120 KCl, 20 KOH, 4 MgCl_2_, 5 EGTA, 0.25 CaCl_2_, 10 HEPES, and 5 Na_2_ATP (325 mOsm, pH adjusted to 7.2 using NaOH). Channelrhodopsin was excited by a 1 or 5 Hz train of blue light pulse (3 ms) delivered with a blue light-emitting diode (LED) source (Thorlabs, M00552407, Newton, NJ, United States). Light switch was triggered by TTL signals from a HEKA EPC-10 double amplifier. All experiments were carried out at room temperature (20–22°C).

### Statistical Analysis

Data analysis and graphing were performed using Excel (Microsoft), Igor Pro (Wavemetrics), Prism (GraphPad), and Clampfit (Molecular Devices). In this study, *n* refers to the number of recordings (one cell per animal). *P* < 0.05 was regarded as statistically significant.

## Data Availability Statement

The raw data supporting the conclusions of this article will be made available by the authors, without undue reservation.

## Author Contributions

YZ and JL designed the experiments and wrote the manuscript. YZ performed the electrophysiology recording. XC, ZS, and ZY carried out the confocal imaging. YL conducted the worm behavioral analysis. All authors contributed to the article and approved the submitted version.

## Conflict of Interest

The authors declare that the research was conducted in the absence of any commercial or financial relationships that could be construed as a potential conflict of interest. The handling editor declared a past co-authorship with one of the author JL.

## Publisher’s Note

All claims expressed in this article are solely those of the authors and do not necessarily represent those of their affiliated organizations, or those of the publisher, the editors and the reviewers. Any product that may be evaluated in this article, or claim that may be made by its manufacturer, is not guaranteed or endorsed by the publisher.

## Supplementary Material

Supplementary Figure 1 A working model of sex-specific neurotransmission at the NMJs in adult *C. elegans*. Adult males process more presynaptic cholinergic vesicle at NMJs and ACh receptors in muscle membrane compared to hermaphrodites. The Cav3 calcium channel CCA-1 plays male-specific roles in spontaneous neurotransmitter release at NMJs. Sex-specific characterizations in both presynaptic neurons and postsynaptic muscles work together to generate sex-specific locomotion in adult *C. elegans*.
