# Supplementary figures and images for: Sexually Dimorphic Neurotransmitter Release at the Neuromuscular Junction in Adult Caenorhabditis elegans
Source: Front Mol Neurosci. 2022 Jan 31;14:780396. doi: 10.3389/fnmol.2021.780396 (PMC8841764; doi:10.3389/fnmol.2021.780396)

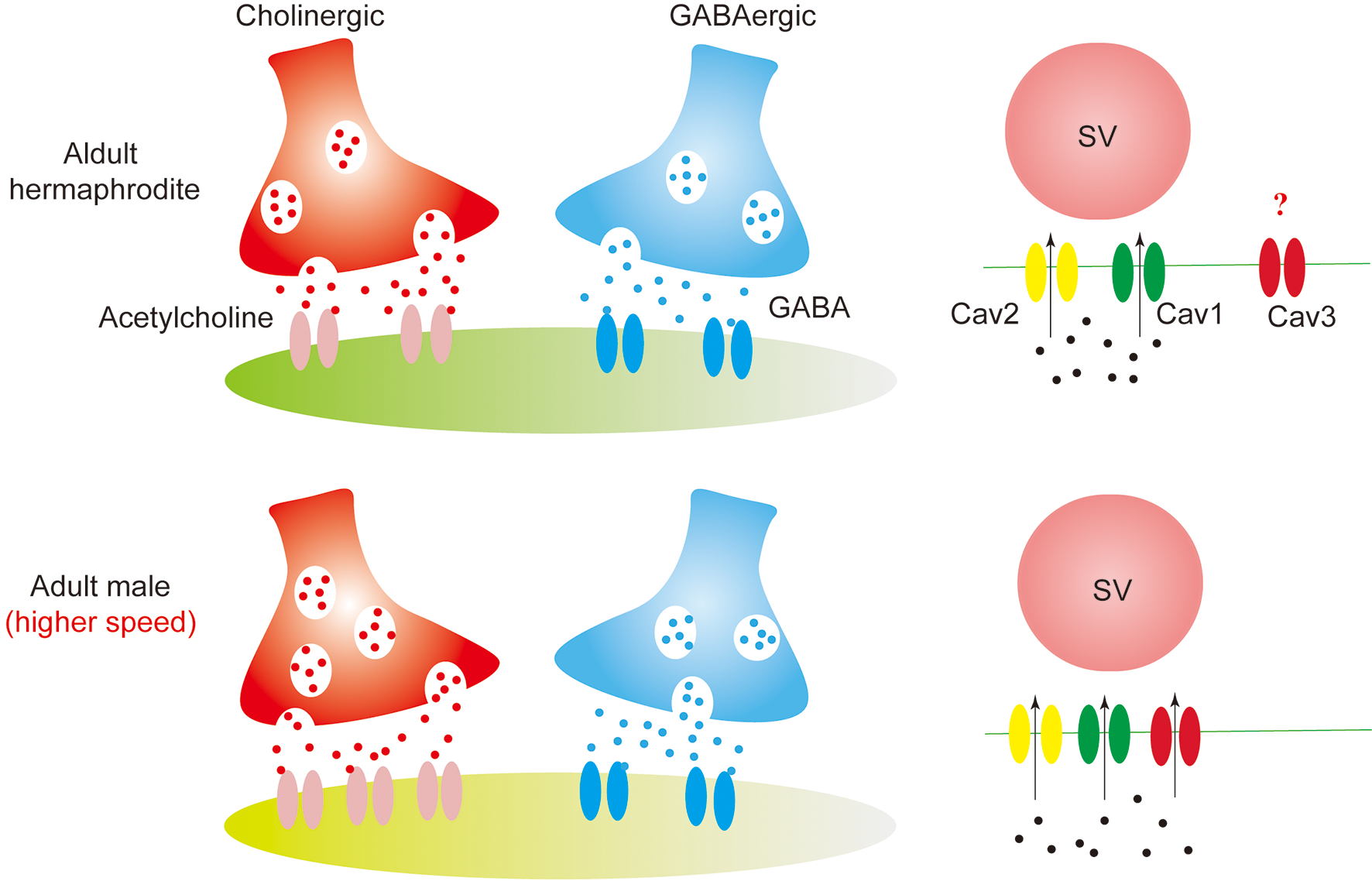

Supplement: Supplementary Figure 1 — A working model of sex-specific neurotransmission at the NMJs in adult C. elegans. Adult males process more presynaptic cholinergic vesicle at NMJs and ACh receptors in muscle membrane compared to hermaphrodites. The Cav3 calcium channel CCA-1 plays male-specific roles in spontaneous neurotransmitter release at NMJs. Sex-specific characterizations in both presynaptic neurons and postsynaptic muscles work together to generate sex-specific locomotion in adult C. elegans. [file Image_1.tif]
